# Knowledge about Obstetric Danger Signs among Pregnant Women in Aleta Wondo District, Sidama Zone, Southern Ethiopia

**DOI:** 10.4314/ejhs.v20i1.69428

**Published:** 2010-03

**Authors:** Mesay Hailu, Abebe Gebremariam, Fessahaye Alemseged

**Affiliations:** 1 Sidam Zone Health Department, SNNPRS, Hawassa, Ethiopia; 2 Department of Population and Family Health, Jimma University, Ethiopia; 3 Department of Biostatistics and Epidemiology, Jimma University, Ethiopia

**Keywords:** Danger signs, Pregnancy, Childbirth, Postpartum, Obstetric care

## Abstract

**Background:**

Raising awareness of women on danger signs of pregnancy, childbirth and the postpartum period is crucial for safe motherhood. In Ethiopia, a country where maternal morbidity and mortality is high little is known about knowledge level of pregnant women on obstetric danger signs. The objective of this study was to assess pregnant women's knowledge about obstetric danger signs in Aleta Wondo district, Sidama Zone, South Ethiopia.

**Methods:**

A community based cross-sectional study was conducted from January 18 – February 20, 2007, on a sample of 812 pregnant women selected from, 8 rural and 2 urban Kebeles. A structured pre-tested questionnaire was used to collect quantitative data on socio-demographic characteristics, obstetric history, and knowledge about danger signs of pregnancy, childbirth and post partum period. Qualitative data was collected through focus group discussion with pregnant women and in-depth interview with traditional birth attendants. The collected data were analyzed using SPSS for Windows version 12.0.1.

**Results:**

Seven hundred forty three pregnant women participated in the study making a response rate of 92%. Out of the 743 pregnant women who participated in this study 226 (30.4%), 305(41.3%) and 279(37.7%) knew at least two danger signs during pregnancy, childbirth and postpartum period, respectively. Being urban resident was consistently found to be strongly associated with mentioning at least two danger signs of pregnancy (OR=4.1; 95% CI: 2.4, 7.0), child birth (OR=3.3; 95% CI: 1.8, 6.1), and postpartum period (OR=8.4; 95% CI: 4.5, 15.4).

**Conclusion:**

This study indicated that the knowledge level of pregnant women about obstetric danger signs (during pregnancy, childbirth and postpartum period) was low and affected by residential area. Therefore, the identified deficiencies in awareness should be addressed through maternal and child health services by designing an appropriate strategies including provision of targeted information, education and communication.

## Introduction

World Health Organization (WHO) estimates that about 300 million women in the developing countries suffer from short and long-term illnesses due to complications related to pregnancy and childbirth. About 529,000 mothers die each year from maternal causes, out of which 99% of deaths being from the developing world. As literatures indicate about 75% of maternal deaths are due to direct obstetric complications such as hemorrhage, sepsis, hypertensive disorders of pregnancy, obstructed and prolonged labor, and unsafe abortion (1–6). In Ethiopia, the levels of maternal mortality and morbidity are among the highest in the world and the current estimate of MMR is 673 per 100, 000 live births and it is reported that Maternal deaths accounted for 21% of all deaths (7).

Maternal morbidly and mortality could be prevented significantly if women and their families recognize obstetric danger signs and promptly seek health care. The commonest danger signs during pregnancy include severe vaginal bleeding, swollen hands/face and blurred vision. Key danger signs during labor and childbirth include severe vaginal bleeding, prolonged labor, convulsions, and retained placenta. Danger signs during the postpartum period include severe bleeding following childbirth, loss of consciousness after childbirth, and fever. Raising awareness of pregnant women on the danger signs would improve early detection of problems and reduces the delay in deciding to seek obstetric care (8, 9).

The national reproductive strategy of Ethiopia has given emphasis to maternal and newborn health so as to reduce the high maternal and neonatal mortality. The strategy focuses on the need to empower women, men families and communities to recognize pregnancy related risks, and to take responsibility for developing and implementing appropriate response to them. One of the targets in the strategies is to ensure that 80% of all families recognize at least three danger signs associated with pregnancy related complications by 2010 in areas where health extension program is fully implemented (10).

Despite the fact that emphasis is given by the national strategy to raise knowledge of obstetric danger signs little is known about the current level of knowledge and the influencing factors in Ethiopia. This study therefore aims to fill this gap by assessing the current status of knowledge of danger signs among pregnant women in the study area.

## Methods and Subjects

This study was conducted from February 18 to March 20, 2007 in Aleta Wondo District which is located 333kms Southeast of Addis Ababa. Aleta Wondo is one of 19 Districts in Sidama Zone of South Nations and Nationalities Regional State. As projected from the 1994 Ethiopian census, the district had a total population of 212,459. Administratively, the District is subdivided into 27 rural and 4 urban Kebeles in which 184,015 and 28,444 dwellers inhabit, respectively (11). According to the report from the district health office there were 1 health center, 2 upgrading health centers, 2 medium private clinics and two non-governmental clinics which provide reproductive health services among others. The study employed community-based cross-sectional design using both quantitative and qualitative methods. All pregnant women with gestational age of at least 3 months were considered as targets for the study. Taking an estimate of pregnant women for the region (4.3%), the total number of pregnant women in the district was presumed to be 9050 (12).

A sample size of 812 was determined using sample size formula for estimating a single population proportion with the assumption that the proportion of pregnant women who know obstetric danger signs, margin of error, confidence interval, design effect and expected non-response rate to be 50%, 5%, 95%, 2 and 10%, respectively. A total of four focus group discussions with average number of 8 discussants per group were conducted on elderly women, pregnant women and married men. In-depth interviews were held with six Traditioal Birth Attendants (TBAs).

Multistage sampling was used to select study subjects. First, all the Kebeles in the District were stratified in to urban and rural. Then 2 out of 4 urban and 8 out of 27 rural Kebeles were randomly selected. The calculated sample size was proportionally allocated to urban (n=106) and rural (n=706) areas respectively. Then census was conducted to register all pregnant women and their gestational age. The number of eligible pregnant women obtained during the census was close to the determined sample size with sampling interval close to one. Therefore, all eligible pregnant women in the selected Kebeles were included in the study. A pre-tested structured questionnaire which was translated to local language (Sidama) was used for interview. The questionnaire included questions on pregnant mothers' socio demographic characteristics, obstetric history and knowledge about danger signs during pregnancy, childbirth and post partum period.

Census was conducted by fifteen trained community health workers (CHA) who can speak local language. Then data was collected by ten trained health extension workers who were supervised by two nurses. Focus group discussions (FDG) were conducted by one MPH and one BSc registered nurse who speak local language. In-depth interview was conducted by one registered BSc nurse. The collected quantitative data were coded, entered, and cleaned, and analyzed using SPSS for Windows version 12.0.1. Analysis of quantitative data was done sequentially starting with univariate using descriptive techniques, then bivariate analysis using chi-square test and finally and logistic regression analysis was done to control for possible con founders. Pregnant women who spontaneously mentioned at least two danger signs out of at least nine danger signs of pregnancy, at least two out of at least seven danger signs during labor and child birth, and at least two out of at least nine danger signs in postpartal period were considered as knowledgeable for the respective category. And those who didn't mention two danger signs were considered as not knowledgeable. Thematic framework analysis was performed for qualitative data analysis. Ethical approval was obtained from Jimma University research and publication office and permission was obtained from the local authorities before undertaking the study. Verbal informed consent was obtained from the study participants before data collection and the collected individual data was kept confidential.

## Results

Out of the sampled 812 pregnant women, 743 were interviewed making a response rate of 92%. The mean age of respondents was 25 ± 4 years.

Majority of the respondents accounting for 644 (86.7%) were rural dwellers, 715 (96.2%) currently in marital union, 682 (91.8%) Sidama by ethnicity, 645 (86.8%), protestant 371 (50.1%) read and write and 710 (95.6%) were housewives. Out of the total respondents only 568 (76.7%) volunteered to disclose their income and out of which 360 (68.7%) had a monthly income of greater than Birr 90. Regarding obstetric history majority 135 (18.2%) were primigravidae, 127 (17.1%) had given birth to one child, 377 (50.7%) had given birth to two to four children and 104 (14.1%) had delivered five or more children (Table 1).

**Table 1:** Socio-demographic and obstetric characteristics of pregnant women, Aleta Wondo Woreda, Sidama Zone, February 18, 2007 to March 20, 2007.

Baseline characteristics	Number	Percent
**Age in years**		
<20	180	24.2
21–25	238	32.0
26–30	271	36.5
>30	54	7.3
**Marital status**		
Currently in marital union	715	96.2
Currently not in marital union	28	3.8
**Residence**		
Urban	99	13.3
Rural	644	86.7
**Religion**		
Protestant	645	86.8
Orthodox	44	5.9
Catholic	29	3.9
Others*	25	3.3
**Educational level**		
Can't read and write	371	49.9
Read and write	105	14.1
Grade 1–4	96	12.9
Grade 5–8	137	18.4
9^th^ grade and above	34	4.6
**Occupation**		
House wife	710	95.6
Gov't employee	12	1.6
House maid	11	1.5
Others**	10	1.3
**Ethnicity**		
Sidama	682	91.8
Amhara	45	6.1
Others***	16	2.1
**Parity**		
Primigravida	135	18.2
Primi-para (1)	127	17.1
Multipara (2–4)	377	50.7
Grandmultipara (5+)	104	14.0
**Monthly income**		
≤90 Birr	178	31.3
>90 Birr	390	68.7

Other* Muslim, traditional, ** occupations like farmer, student, self employee, and jobless Others*** Guraghe,Tigray, and Silite

When asked to mention danger signs during pregnancy the most common spontaneously mentioned danger signs were vaginal bleeding by 341 (45.9%), difficulty of breathing by 105 (14.1%) and loss of consciousness by 94 (12.7%). Other signs mentioned include high fever accounting for 68 (9.2%), severe headache for 55 (7.4%), and severe abdominal pain for 52 (7.0%). Two hundred ninety (39.0%) didn't know any danger signs of pregnancy (Table 2). Two hundred twenty six (30.4%) mentioned at least two danger signs during pregnancy and 314 (63.6%) believed that a woman could die of the above mentioned danger signs. In line with the above report,

Most of the focus group discussants and in-depth interview participants mentioned decreased fetal movement, weight loss, fever, Weakness, dizziness, severe abdominal pain, vaginal bleeding and malposition as the danger signs during pregnancy.

**Table 2:** Knowledge of danger signs of pregnancy among pregnant women in Aleta Wondo district, Sidama Zone, February 18 to March 20, 2007.

Danger signs of pregnancy	Frequency	
	Number	Percent
Bleeding	341	45.9
Difficulty of breathing	105	14.1
Loss of consciousness	94	12.7
High fever	68	9.2
Sever headache	55	7.4
Sever abdominal pain	52	7.0
Blurred vision/dizziness	50	6.7
Convulsion	35	4.7
Swollen hand /face	24	3.2
Don't know	290	39

The most commonly mentioned danger signs of labor and childbirth were excessive bleeding by 409 (55%), placenta not delivered within 30 minutes after delivery of baby by 382 (51.4%), labor lasting more than 12 hours by 321 (43.2%), and loss of consciousness by 81 (10.9%) of the respondents (Table 3). Three hundred five (41.3%) of respondents mentioned at least two danger signs during pregnancy and 516 (69.4%) believed that a woman could die of the mentioned problems. *Danger signs mentioned by most of focus group discussants during labor and childbirth were labor lasting more than one day, retained placenta, bleeding and inability to push the baby.*

**Table 3:** Number and percentage of pregnant women who mentioned danger signs during labor and child birth in Aleta Wondo district, Sidama Zone, March, 2007.

Danger signs during labor and child birth	Number	Percent
**Severe bleeding**	409	55.0
**Retained placenta**	382	51.4
**Laborlasting>12 hrs**	321	43.2
**Loss of consciousness**	81	10.9
**Sever head ache**	52	7.0
**High fever**	41	5.5
**Convulsions**	32	4.3
**malposition/presentation**	21	2.8
**Don't know**	143	19.2

The danger signs of post partum period commonly mentioned include severe bleeding by 438 (59%), difficulty of breathing by 133 (17.9%), loss of consciousness by 107 (14.4%), and extreme weakness by 103 (13.9%) of the respondents (Table 4). Two hundred seventy nine (37.7%) of the pregnant women mentioned at least two serious danger signs during post partum period. *Most of the focus group discussants also mentioned excessive bleeding, severe weakness, dizziness, abdominal pain/ cramps as danger signs after delivery of baby.*

**Table 4:** Post-partal period danger signs mentioned by currently pregnant women in Aleta Wondo district, Sidama Zone, March 2007

Danger signs in postpartal period	Number	Percent
Severe bleeding	438	59
Difficulty breathing	133	17.9
Loss of consciousness	107	14.4
Severe weakness	103	13.9
Convulsion	85	11.4
Malodorous vaginal discharge	84	11.3
Sever headache	80	10.8
Visual disturbance	69	9.3
Swollen hands/face	15	2.0
Uterine prolapse	7	0.9

Danger signs of serious health problems of newborn commonly reported were fast or difficult breathing by 367 (49.4%), poor sucking or feeding by 308 (41.5%) and pus or discharge from umbilical cord by 146 (19.7%) of the respondents. Basic cares of newborn mentioned by the respondents in order of their frequency were cord care by 535 (72%), exclusive breast-feeding by 458 (61.6%), dry and wrap by 376 (50.6%), and eye care by 45 (6.1%) (Table 5).

**Table 5:** Knowledge of newborn care by pregnant women in Aleta Wondo district, Sidama Zone, March 2007.

Types of care	Number	Percent
Cord care	535	72.0
Exclusive breast feeding	458	61.6
Dry and wrap	376	50.6
Washing immediately	172	23.1
Eye care	45	6.1
Didn't know	19	2.6

In the binary logistic regression analysis, urban residence (OR=4.1; 95% CI: 2.4, 7.0), being in current marital union (OR=31.9; 95% CI: 6.6, 153.7) and having attended high school and above (OR=2.8; 95% CI: 1.1, 6.7) were independently associated with mentioning of at least two danger signs of pregnancy. The variables that were independently associated with mentioning of at least two danger signs during labor were urban residence (OR=3.3; 95% CI: 1.8, 6.1), being currently married (OR=6.4; 95% CI: 2.4, 16.6) and multiparity (OR for parity 3 to 5=2.7; 95% CI: 1.3, 5.9 and OR for parity 6 or more=2.5; 95% CI: 1.2, 5.2). Whereas it was only urban residence which was independently associated (OR=8.4; 95% CI: 4.5, 15.4) with mentioning of at least two danger signs in post-partal period (Table 6).

**Table 6:** Association between knowledge of at least 2 danger signs during pregnancy, labour and postpartal period and selected socio demographic characteristics of pregnant women, Alta Wondo, Sidama District Zone, March 2007.

Characteristics		Adjusted OR* (95% CI) for knowledge about danger signs of pregnancy	Adjusted OR (95%CI) for knowledge about danger signs during labor	Adjusted OR (95%CI) for knowledge about danger signs in postnatal period
**Age**				
	<20	1	1	1
	20–24	0.6(0.34, 1.2)	1.4(0.8,2.4)	0.8(0.4,1.5)
	25–29	0.757(0.4, 1.4)	2.0(1.1,3.7)	1.0(0.5,1.9)
	30–34	1.6(0.8, 3.4)	2.8(1.4,5.7)	1.3(0.6,2.7)
	>=35	1.8(0.6, 5.1)	4.3(1.5,12.6)	2.0(0.7,5.7)
**Residence**				
	Urban	4.1(2.4, 7.0)	3.3(1.8–6.1)	8.4(4.5–15.4)
	Rural	1	1	1
**Current marital** **status**				
	Married	31.9(6.6–153.7)	6.4(2.4–16.6)	14.2(0.6–43.8)
	Not married	1	1	1
**Educational level**				
	Illiterate	1	1	1
	Read & write	1.4(0.8–2.2)	1.3(0.8–2.1)	1.8(1.1–2.8)
	1–4	1.0(0.9–1.8)	0.98(0.6–1.6)	0.9(0.5–1.4)
	5–8	1.3(0.6–2.1)	1.1(0.7–1.5)	0.7(0.4–1.1)
	9^th^ and above	2.8(1.1–6.7)	1.6(0.7–3.9)	0.7(0.3–1.8)
**Parity**				
	0–2	1	1	1
	3–5	1.3(0.6–2.9)	2.7(1.3–5.9)	1.2(0.5–2.7)
	>=6	1.2(0.5–2.6)	2.5(1.2–5.2)	1.9(0.8–4.2)

* The OR is adjusted for mutual effect of the variables on each other.

## Discussion

Knowledge of danger signs of obstetric complications during pregnancy, labour and postnatal period is the first essential step for appropriate and timely referral. The findings of this study has provided an insight information on pregnant women's knowledge about obstetric danger signs in the study area, which could help in designing appropriate interventions and as a base for further wide scale studies in other part of the country.

In this study about half (45.9%) of the study subjects mentioned vaginal bleeding as danger sign during pregnancy, which is higher than the findings in Burkina Faso (39.4%) and Guatemala (31.0%) (13, 14). This difference might be due to socio-cultural difference and difference in implementation of relevant health intervention programs.

There is evidence that show the major causes of maternal mortality to be hemorrhage, sepsis, and hypertensive disorder of pregnancy and pregnant mothers need to have adequate knowledge about the signs indicating these problems (15). One third of the (30.9%) of respondents mentioned at least two danger signs of pregnancy. The fact that large proportion of pregnant women did not know danger signs of serious health problems could adversely affect their preparedness for pregnancy complications.

Key danger signs during labor and childbirth are severe vaginal bleeding, prolonged labor, convulsions, and retained placenta. In this study a proportion of pregnant women, who mentioned bleeding as danger signs during labor and childbirth (55.0%) were higher than the Kenyan study finding (37.0%) (15). The proportion of the study subjects who mentioned labor lasting more than 12 hours as danger sign (43.2%) was lower than Burkina Faso's study finding (82.2%) but higher than Guatemala's study finding (11.0%) (13, 14). This difference might be due to difference in socioeconomic and health intervention activities in the areas.

It is believed that if pregnant women and their family recognize danger signs of obstetric complications they may seek care and it can reduce first delay to seek health service (16). This study showed that only less than half of pregnant women could mention at least two danger signs during labor and childbirth. The rate of death in this period is higher than for any other 36–48 hour period during the nine-months of pregnancy and 42-days postpartum (5). Hence, efforts should be exerted to increase awareness on danger signs during labor and childbirth.

Danger signs important to identify during the postpartum period include severe bleeding following childbirth, loss of consciousness after childbirth, and fever. Post-partum hemorrhage is a leading cause of approximately 30% maternal deaths worldwide (17). In this study more than half of the studied pregnant women mentioned bleeding as danger sign during postpartum period which is higher as compared with the finding in Guatemala (28.0%) and Indonesia (29.0%) (13, 18). These differences in knowledge level could again be due to a difference in sociodemographic, cultural, and health interventions as well as methodological difference. Only 37.7% of women know at least two danger signs during post partum period in this study, indicating that large proportion of pregnant women didn't know danger signs during post partum period. Hence concerned effort should be exerted to increase their awareness.

Being urban resident was found to have a significant association with mentioning at least two danger signs during pregnancy, child birth and postpartum period. This could be due to the fact that urban residents have better access to health information and maternal health services as compared with rural counterparts (19). Those who were currently married were likely to be knowledgeable on danger signs of pregnancy and child birth. This needs to be further investigated with robust analytical studies.

In general, this study showed that significant proportion of the pregnant women were unaware of obstetric danger signs. This indicates the large proportions of pregnant women who do not have the knowledge are likely to delay in deciding to seek care. Therefore, the MOH, regional health bureau, zonal health department, Woreda health office as well as other partner organizations that are working in areas of reproductive health should design appropriate strategies including provision of targeted health education or provide information, education and communication to pregnant mothers to increase their awareness and thus enable early recognition of serious health problems during pregnancy, labor and post-partal period.
